# Pharmacist-participated medication review in different practice settings: Service or intervention? An overview of systematic reviews

**DOI:** 10.1371/journal.pone.0210312

**Published:** 2019-01-10

**Authors:** Rafaella de Oliveira Santos Silva, Luana Andrade Macêdo, Genival Araújo dos Santos, Patrícia Melo Aguiar, Divaldo Pereira de Lyra

**Affiliations:** 1 Laboratory of Teaching and Research in Social Pharmacy (LEPFS), Department of Pharmacy, Federal University of Sergipe, São Cristóvão, Sergipe, Brazil; 2 Department of Pharmacy, Faculty of Pharmaceutical Sciences, University of São Paulo, São Paulo, Brazil; University of Oxford, UNITED KINGDOM

## Abstract

**Introduction:**

Medication review (MR) is a pharmacy practice conducted in different settings that has a positive impact on patient health outcomes. In this context, systematic reviews on MR have restricted the assessment of this practice using criteria such as methodological quality, practice settings, and patient outcomes. Therefore, expanding research on this subject is necessary to facilitate the understanding of the effectiveness of MR and the comparison of its results.

**Aim:**

To examine the panorama of systematic reviews on pharmacist-participated MR in different practice settings.

**Methods:**

A literature search was undertaken in Biblioteca Virtual em Saúde (BVS), Embase, PubMed, Scopus, The Cochrane Library, and Web of Science databases through January 2018 using keywords for "medication review", "systematic review", and "pharmacist". Two independents investigators screened titles, abstracts, full texts; assessed methodological quality; and, extracted data from the included reviews.

**Results:**

Seventeen systematic reviews were included, of which sixteen presented low to moderate methodological quality. Most of reviews were conducted in Europe (n = 7), included controlled primary studies (n = 16), elderly patients (n = 9), and long-term care facilities (n = 8). Seven reviews addressed MR as an intervention and thirteen reviews cited collaboration between physicians and pharmacists in the practice of MR. In addition, thirteen terminologies for MR were used and the main objective was to identify and solve drug-related problems and/or optimize the drug use (n = 11).

**Conclusion:**

There is considerable heterogeneity in practice settings, population, definitions, terminologies, and approach of MR as well as poor description of patient care process in the systematic reviews. These facts may limit the comparison, summarization and understanding of the results of MR. Furthermore, the methodological quality of most systematic reviews was below ideal. Thus, international agreement on the MR process is necessary to assess, compare and optimize the quality of care provided.

## Introduction

Medication Review (MR) has been defined as a structured assessment of patients’ pharmacotherapy whose aim is to optimize the drug use and to improve health outcomes [1]. Despite that concept, MR is used as a generic term and its practice can be performed by some health professionals, such as physicians, nurses and pharmacists, causing confusion regarding its characterization and effectiveness. In the MR, pharmacists play an important role in the detection and resolution of drug-related problems (DRPs) through interaction with patients and/or healthcare professionals [2].

The MR conducted by pharmacists may be classified into three types: Prescription Review, Concordance and Compliance Review (Adherence Support Review), and Clinical Medication Review (with or without prescribing) [3, 4]. This practice, the last type particularly, must be conducted in collaboration with health professionals [5]. There are models of MR performed by pharmacists with collaboration of other health professionals in countries where pharmaceutical education is clinic-oriented as well as in those where pharmacists have no formal clinical education [6–11].

In Australia, there are examples of MR in which after the pharmacist assesses the patient’s pharmacotherapy, he sends a report with recommendations to the patient’s physician. After agreement with the pharmacist, the physician discusses the proposed recommendations with the patient [8, 9]. The implementation of these recommendations made by the pharmacist may be influenced by some factors such as: a good working relationship between the pharmacist and the health care team [2], the type of communication between the pharmacist and the team (verbal or written) [2, 10, 11] and the clinical relevance of the recommendations [10, 11].

In this context, studies show that pharmacist-participated MR has a positive impact on patients in practice settings such as community pharmacies [12, 13], long-term care facilities [14,15], outpatient clinic [16], home care [17, 18] and hospitals [19, 20]. Besides the identification and resolutions of DRPs [21–23], the studies highlight benefits such as increase in quality of life [24], decrease of hospitalizations and health costs [25, 26]. In order to achieve such results, the implementation of MR demands changes in pharmacists’ professional and social behaviour [18].

Although a previous overview of systematic reviews has corroborated the importance of MR for the improvement of patients’ health outcomes, it restricted relevant aspects such as methodological quality, practice settings, and assessed outcomes [27]. Moreover, the mentioned overview didn’t focus on concepts, terminologies and approach (as service or practice component) of MR as well as the professionals involved in this practice (interprofessional collaboration). The study of these variables is necessary to facilitate the understanding of the effectiveness of MR and to compare results.

Taking this into consideration, the present overview aimed to examine: 1) the panorama of systematic reviews on pharmacist-participated MR in different practice settings; 2) methodological quality of systematic reviews included in this overview; 3) the concepts, terminologies, and MR approach as well as the interprofessional collaboration in MR.

## Methods

This overview of systematic reviews was performed according to the tool “A MeaSurement Tool to Assess systematic Reviews” (AMSTAR) [28].

### Definitions

This overview of systematics reviews adopted the following concepts:

- Systematic reviews: studies that: (i) present a clear research question and/or eligibility criteria used to select primary studies; (ii) describe all information sources and the keywords used in the search; (iii) present the number of primary studies found in the information sources and included in the final sample of systematic review.
- Medication Review: critical and structured assessment of patients’ drugs with the objective of coming to an agreement of their pharmacotherapy, improving treatment, decreasing DRPs and costs with healthcare systems [29]. MR can be classified in Prescription Review, Concordance and Compliance Review (Adherence Support Review), and Clinical Medication Review (with or without prescribing) depending on their objective, patient’s presence, access to information and drugs and/or patient’s clinical conditions [3, 4],

### Literature search

A comprehensive literature search was conducted on the following databases: Biblioteca Virtual em Saúde (BVS), Embase, PubMed, Scopus, The Cochrane Library, and Web of Science for systematic reviews with or without meta-analysis published until 31 January 2018. To that end, Medical Subject Headings (MESH) vocabulary [30] and non-standard terms (text words) were used. Full search strategy can be seen in S1 Table. This overview has not been registered on PROSPERO International prospective register of systematic reviews.

### Systematic reviews selection

Systematic reviews were selected in four stages: 1) exclusion of repeated articles; 2) title and abstract screening; 3) full-text screening; and, 4) manual screening of references of the systematic reviews included after reading full articles. Systematic reviews were independently selected by two investigators (R.O.S.S. and L.A.M.) and divergences were solved by a third investigator (G.A.S.J.). If articles were not available in full, authors were contacted via ResearchGate (www.researchgate.net) and e-mail. The stages 1, 2, and 3 of the study selection were performed using the Rayyan QCRI tool [31].

The included systematic reviews attended the following criteria: (i) they were published in English, Portuguese or Spanish; (ii) they were systematic reviews followed or not by meta-analysis; (iii) they examined MR (focused on MR or included different pharmaceutical services/interventions, but the results were presented by type of service/intervention); (iv) they adopted terminology for MR adopted in the search strategy; and (vi) pharmacist-participated MR in all primary studies with or without collaboration of other health professionals. Systematic reviews that did not present the definition of MR were included only if the interventions described in primary studies accorded to the concept of MR adopted.

In this overview, other systematic reviews were excluded because: i) full text was unavailable; (ii) MR was performed collaboratively by pharmacist and other health professionals but the pharmacist’s role within the team was not clearly defined in the primary studies included.

### Data extraction

Two investigators (R.O.S.S. and L.A.M.) extracted independently the following data: authors, publication year, main author’s country, aim, country of primary studies, study design, practice setting, and population, number of primary studies included in the systematic review and meta-analysis, number of primary studies related to MR, assessed outcomes related to the drug use process, and economic, clinical, and human outcomes (ECHO model) [32], main results, concepts, terminologies, and approach (service or intervention) of MR, interprofessional collaboration, structure, processes, and outcomes variables [33, 34] described in the systematic reviews as well as limitations or biases. Except for the number of primary studies included in the systematic reviews and meta-analysis, all data were extracted only from primary studies on MR. In case of data absence or clear pieces of information, it was considered that authors did not report such variable. Discrepancies among investigators were solved by consensus.

Study design, practice setting and population were determined according to the authors of systematic reviews. Regarding terminologies of MR, the words used in the search strategies of the included systematic reviews were not considered. If the review presented different MR definitions, the one presented in the methodology was adopted. Moreover, in the absence of a clear definition, components or objectives of MR were extracted. Interprofessional collaboration was considered present if at least one primary study reported it.

### Quality assessment

Two investigators (R.O.S.S. and L.A.M.) analyzed independently the methodological quality, and discrepancies were solved by consensus. To achieve that, the AMSTAR tool [28] was used, which is composed by 11 criteria, each one judged as “yes,” “no,” “cannot answer,” or “not applicable”. Total score was obtained by the attribution of one point to “yes” answers and zero to other answers, varying score from 0 to 11. The score was ranged according to Mikton and Butchart (2009) [35]: i) score 0–4, low quality; ii) 5–8, moderate quality; and, iii) 9–11, high quality.

### Agreement between investigators

Cohen’s Kappa index (k) was used to measure degree of agreement between the two investigators (R.O.S.S. and L.A.M.) in the title, abstract and full text screenings as well as in the assessment of methodological quality. Degree of agreement was stratified: i) k < 0.10, no agreement; ii) k < 0.40, weak agreement; iii) 0.40 < k < 0.75, good agreement; and, iv) k > 0.75, excellent agreement [36].

## Results

### Selection of systematic reviews

The literature search identified 3,053 articles, from which 2,950 were excluded mainly because of: i) simultaneous indexation in two or more databases; ii) language; iii) they were not a systematic review; and/or, iv) they did not examine MR. Thus, 103 articles were selected to full-text screening, from which 17 reviews met the inclusion criteria. Their references were revised manually, and 68 were identified as potentially relevant. From these, none met the inclusion criteria. Excluded full texts and their reasons for exclusion are summarized in S2 Table.

**Fig 1:**
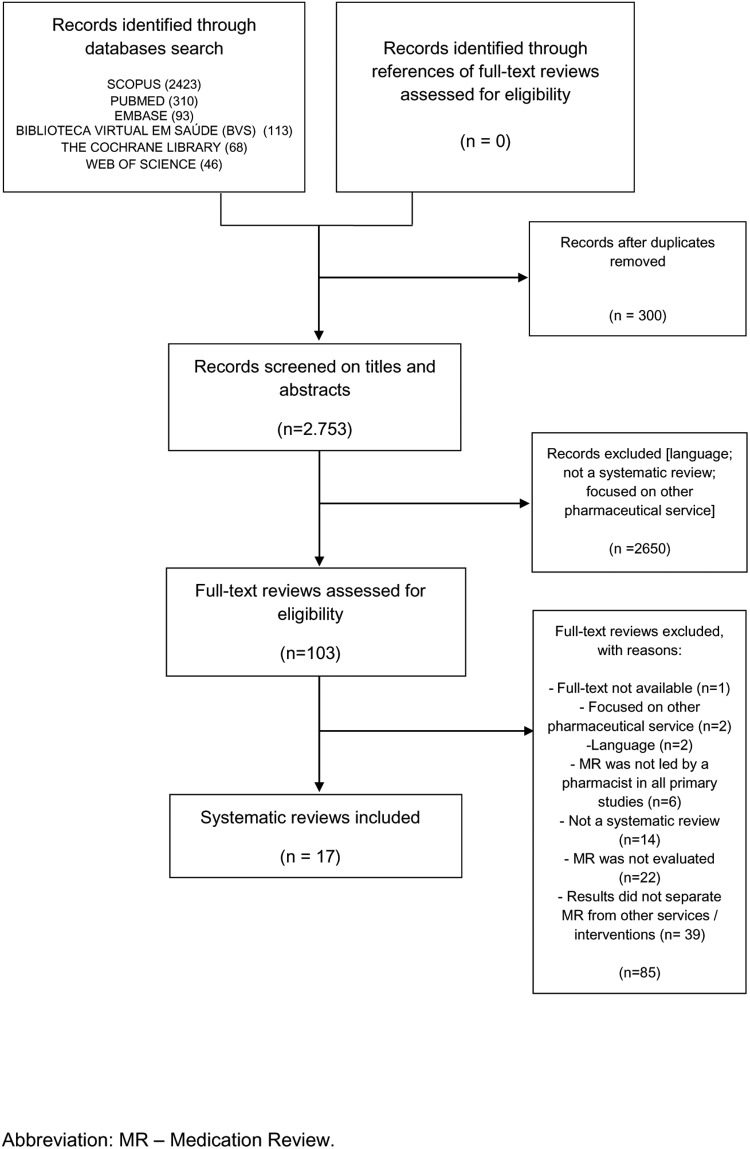
Flowchart of systematic reviews included in this overview.

From 17 articles included, 10 focus on MR [37–46] and seven include other services/interventions besides MR [47–53]. Fig 1 illustrates the selection process.

Degree of agreement between the two investigators (R.O.S.S. and L.A.M.) was excellent for title and abstract screenings (k1 = 0.942) and full-text screening (k2 = 0.805).

### Quality assessment

**Fig 2:**
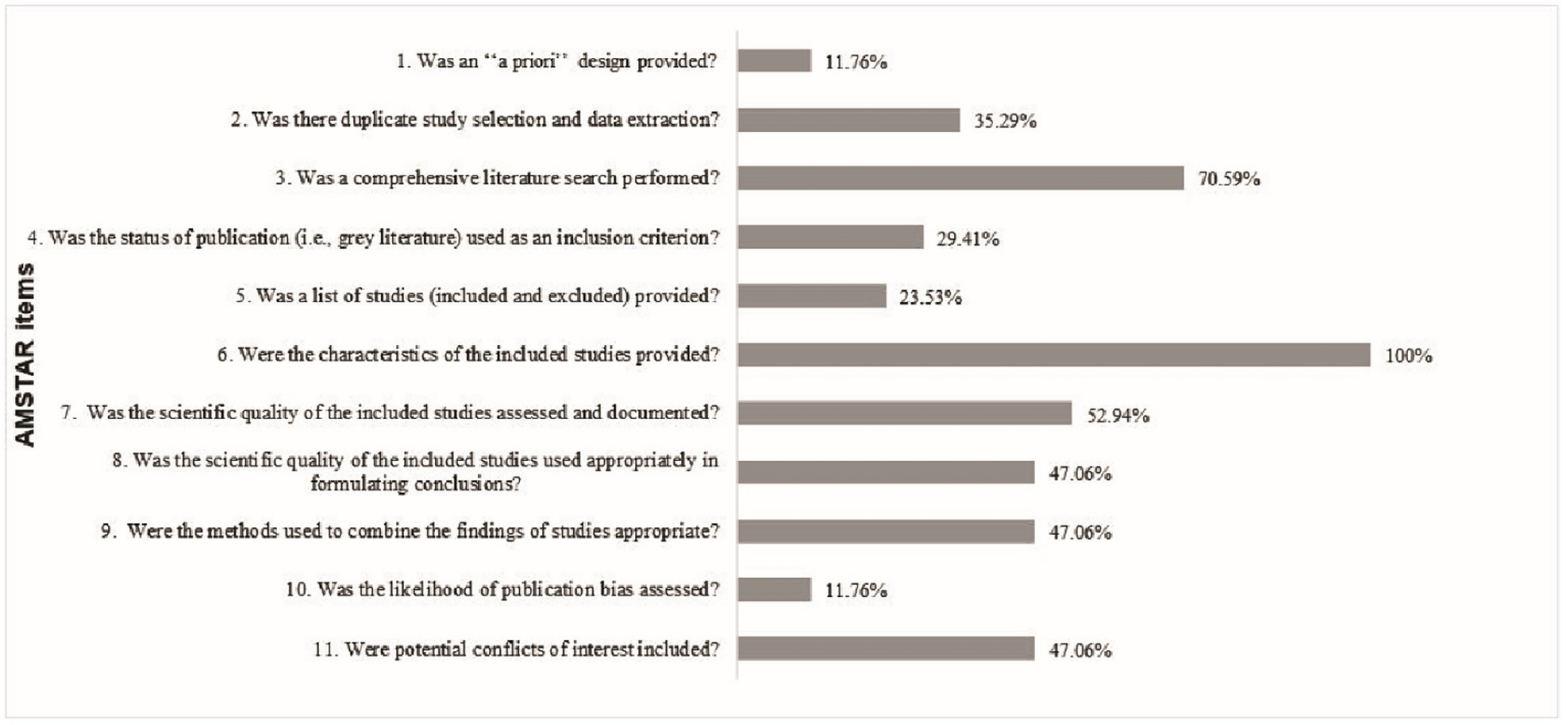
Percentage of systematic reviews that appropriately address each (AMSTAR) item.

Scores of methodological quality after consensus are presented in Table 1. The percentage of reviews that met each item of AMSTAR is presented in Fig 2. Score varied from 2 to 9, presenting average 4.82 ± 2.09. Degree of agreement between the two investigators (R.O.S.S. and L.A.M.) was excellent (k = 0.802). Among the 17 reviews, eight were categorized as low quality [37, 39, 47–49, 51–53]; eight as moderate quality [38, 40–43, 44, 46, 50]; and one review presented high quality [45]. Seven articles that presented from moderate to high quality were published from 2013 to 2017 [40–46]. Seven reviews with meta-analysis presented AMSTAR score between 4–8 [37, 40– 42, 44, 49, 53].

**Table 1:** Description of the systematic reviews’ aim; primary studies’ countries, practice setting, study design and population; and AMSTAR score for quality assessment of systematic reviews followed or not by meta-analyzes.

Reference	Aim	Primary studies countries	Practice setting	Study design	Population	AMSTAR score
[37]	To evaluate systematically and to quantify the effects of medication review by pharmacists on substantive clinical and qualitative outcomes for older people across all care settings	Australia, Canada, Northern Ireland, Singapore, United Kingdom, and United States	Hospital, primary care or clinic, pharmacy, patient’s home, and nursing home	Randomized controlled trial	Older patients (mean age > 60 years) with a range of diseases (more than one diagnostic category)	4
[38]	To identify, assess, and summarize the literature investigating the effect of pharmacist-led medication reviews in hospitalized patients	Australia, Belgium, Canada, Denmark, India, Iran, Israel, Jordan, Northern Ireland, Norway, Oman, Spain, Sweden, United Kingdom, and United States	Hospital setting	Descriptive study and controlled study	Hospitalized patients	8
[39]	To investigate how the extent of collaboration between the GP and the pharmacist impacts on the implementation of recommendations arising from medication review	Australia, Canada, Netherlands, New Zealand, United States, and United Kingdom	Community pharmacy, interdisciplinary health clinic, health centre ambulatory clinic, university, general practice, dispensing general practice, and family practice	Randomized clinical trial	Home-dwelling patients (≥ 70 years) in primary care that was not recently discharged (<1 month)	2
[40]	To evaluate the effectiveness of pharmacist-led medication review in chronic pain management	Canada, Germany, United Kingdom, and United States	University pain clinic, general practice, and community pharmacy	Randomized controlled trial and cluster randomized controlled trial	Adult patients with chronic pain	6
[41]	To examine the impact of fee-for-service pharmacist-led medication review on patient outcomes and quantify this according to the type of review undertaken	Australia, Belgium, Canada, Chile, Denmark, Germany, Netherlands, United Kingdom, and United States	Pharmacy, patients’ home, general practice clinic/surgery, and community health centre	Randomized and non-randomized controlled trial	Adult patients	5
[42]	To summarize the available evidence on the effect of pharmacist-led medication review initiated early within a patient’s hospital course on the length of hospital stay, and on 3-month mortality, hospital readmissions and emergency department revisits based on observed data	Belgium, Canada, Denmark, Northern Ireland, and Sweden	Hospital ward	Randomized controlled trial and controlled clinical trial	Adult patients (>18 years) who presented to an acute care hospital for an unexpected illness	8
[43]	To systematically review the processes and outcomes of clinical medication review in community-settings in Australia	Australia	Community setting	Controlled trial, observational study, uncontrolled study, qualitative study, and survey study	NR	6
[44]	To provide a timely evaluation of the evidence base for pharmacist-provided medication review in the elderly compared with usual care	Australia, Belgium, Brazil, Canada, Denmark, Germany, Malaysia, Netherlands, New Zealand, Northern Ireland, Portugal, Republic of Ireland, Scotland, Spain, Sweden, United Kingdom, and United States	Hospital outpatient clinic, community pharmacy, primary care (such as physician offices), patient’s home, and mixed setting (typically with the first intervention carried out at the hospital, and follow-up conducted by telephone or home visits)	Randomized controlled trial	Community dwelling patients with a mean or median age of at least 65 years	5
[45]	To evaluate the impact of in-hospital pharmacist-led medication reviews on clinical outcomes at different time points	Australia, Belgium, China, Denmark, Ireland, Israel, Northern Ireland, Portugal, Sweden, United Kingdom, and United States	Hospital and care units	Randomized controlled trial	Adult or pediatric patients	9
[46]	To assess the impact of medication reviews in aged care facilities, with additional focus on the types of medication reviews (prescription and/or clinical medication reviews) in a single care setting (aged care homes) using a specific study design (randomized controlled trials and prospective studies)	Australia, Belgium, Netherlands, North Ireland, Singapore, Spain, Sweden, Switzerland, United Kingdom, and United States	Aged care facilities	Randomized controlled trial, nonrandomized controlled trial, and observational study (longitudinal and pre and post intervention)	Older people (mean age of subjects > 60 years)	5
[47]	To specifically evaluate the impact of pharmacist delivered community-based services to optimize the use of medications for mental illness	Australia, Sweden, United Kingdom, and United States	Domiciliary and residential aged care setting	Controlled trials, randomized controlled trials, and cluster randomized controlled trials	Patients with mental illness	4
[48]	To provide descriptions of existing remuneration models for pharmacist clinical care services and to summarize the existing evaluations of economic, clinical, and humanistic outcome studies of the remuneration models	Australia	NR	Multistep assessment interviews, focus research group, and mail survey	Stakeholders, pharmacists, consumers and facilitators	3
[49]	To evaluate the evidence pertaining to the impact of medication reviews and/or educational interventions on psychotropic drug use in long-term care facilities	Australia, Sweden, United Kingdom, and United States	Long-term care facility	Randomized and non-randomized controlled trial	Elderly residents (≥ 65 years) who use antipsychotic or hypnotic drugs	4
[50]	To identify, assess, and summarize available scientific evidence about the effect of interventions that could be used to reduce potentially inappropriate use of drugs in nursing homes	Australia, Sweden, and United Kingdom *	Nursing home	Randomized controlled trial	Elderly patients	5
[51]	To interpret the results of studies that have evaluated any type of strategy to improve prescribing in care homes	Australia and United Kingdom	Care homes settings (nursing homes, residential homes, and mixed homes)	Randomized controlled study, cluster randomized controlled study, and controlled before-and-after study	Care home patients or older people	4
[52]	To introduce the concept of safe medication use to both patients and clinicians by presenting multifaceted pharmaceutical concerns in the prevention of medication-related falls among patients in all settings (community dwelling, nursing home, and hospital)	NR	Community dwelling, nursing home, and hospital	Randomized controlled trials**	Elderly patients	2
[53]	To identify and analyse full economic evaluation studies assessing the cost-effectiveness of PPS in community setting in Europe and to summarize their findings	Spain and United Kingdom	Community setting	Randomized controlled trial and cluster randomized controlled trial	Elderly and patients with chronic pain	2

Abbreviations:

*Does not report the country of all primary studies;

**Does not report the study design of all primary studies;

AMSTAR—A MeaSurement Tool to Assess systematic Reviews; GP—General practitioner; NR—Not reported.

Item six (characteristics about participants, interventions, and results) was presented by all reviews [37–54]. Most of reviews performed literature search in at least two databases and supplementary search (item 3) [37, 38, 42–49, 51, 53] and performed the quality assessment of systematic reviews (item 7) [37, 38, 40–45, 50]. On the other hand, only two reviews registered the protocol for systematic review (item 1) [40, 42] and presented the assessment of publication bias (item 10) [41, 45].

### Characteristics of systematic reviews

Characteristics of systematic reviews are described in Table 1. All reviews were published in English between 2005 and 2016. The main authors of the included systematic reviews were from four continents: America, Asia, Europe, and Oceania. Most of reviews had their main authors from Europe (seven reviews; 41.17%) [30–33, 40, 41, 43, 48], Asia (four reviews; 23.53%) [40, 44, 46, 52], and Oceania (four reviews; 23.53%) [41, 43, 47, 49], especially Australia (three reviews; 17,64%) [43, 47, 49]. Primary studies were performed in 28 different countries, and Australia (n = 13) [37–39, 41, 43–46, 48–51] and the United Kingdom (n = 13) [37, 38, 41, 43–51] were the most frequent countries. No primary study of the systematic reviews was found in Latin America.

Most reviews included controlled studies [37–43, 44–47, 49–53]. Six reviews included only controlled randomized trials [37, 39, 45, 50, 53] and five reviews included primary studies of different design as observational, descriptive, qualitative, surveys, and controlled study [38, 43, 46, 48, 52]. Regarding population, most reviews included elderly patients [37, 39, 44–46, 49–53].

Most frequent primary studies settings were long-term care facilities [37, 46, 47, 49–52], hospitals [37, 38, 42, 44, 45, 52], primary care or clinics [37, 39– 42, 45], pharmacies [37, 39, 40, 41, 44], and patient’s home [37, 41, 44, 47].

### Number of primary studies in the systematic reviews, meta-analysis, and related to MR; assessed outcomes, main results; and structure, processes and outcomes variables

Number of primary studies in the systematic reviews, meta-analysis and related to MR as well as assessed outcomes and main results are presented in Table 2. Number of primary studies included in the reviews varied from 5 [40] to 69 [52]. The minimum and maximum number of primary studies in the systematic reviews related to MR was 4 [44, 51–53] and 63, respectively [43]. Only seven of these reviews performed meta-analysis [37, 40, 41, 44, 45, 47, 49], in which the number of primary studies varied from 3 to 25 [37, 40].

**Table 2:** Number of primary studies in the systematic reviews, meta-analysis, and related to MR; assessed outcomes, main results; and structure, processes and outcomes variables.

Reference	Number of primary studies in the SR	Number of primary studies in the meta-analysis	Number of primary studies related to MR	Assessed outcomes	Main results
[37]	32	Y (25)	32	Primary: proportion of patients with one or more hospital emergency admission (all-cause). Secondary: all-cause mortality and mean drugs prescribed	RT 1- All-cause admission (RR 0.99, 95% CI 0.87 to 1.14, p = 0.92); mortality, (RR 0.96, 95% CI 0.82 to 1.13, p = 0.62); and numbers of drugs prescribed (NDP = -0.48, 95% CI -0.89 to -0.07)
[38]	31	N (0)	31	Data on process and implementation of the pharmaceutical service (number of interventions, acceptance rate and percentage of recommendations implemented by physicians), presence of elements of medication review process and patient outcomes (e.g. health-related quality of life)	RT 2- The number of MRPs varied from 81 to 5122. The proportion of MRPs varied from 0.13 to 10.6 per patient. The acceptance rate of recommendations varied from 39% to 100%. The positive effects were: better quality of prescribing, satisfaction with the pharmacist’s service among patients or personnel; decrease of visits to the emergency department, drug-related readmissions, all-causes readmissions, length of in-hospital stay, and costs. The single negative result was on length of in-hospital stay, while health-related quality of life and overall survival only showed overall non-significant results
[39]	12	N (0)	12	Number of key elements that reflects collaborative aspects between a GP and a pharmacist, implementation rate of recommendations following DRPs identified during medication review	RT 2- The mean number of key elements within the intervention was 5.2 (range 1–8). The mean implementation rate of recommendations was 50% (range 17–86). The association between the number of key elements present in the intervention and the implementation rate of recommendations was significant: β = 0.085 (95% CI 0.052 to 0.128; p<0.0001)
[40]	5	Y (3)	5	Pain intensity, physical functioning, patient satisfaction, quality of life, and adverse effects	RT 1 –A 0.8-point reduction in pain intensity on a 0 to 10 numerical rating scale at 3 months (95% CI, -1.28 to -0.36) and a 0.7-point reduction (95% CI, -1.19 to -0.20) at 6 months; a 4.84-point (95% CI, -7.38 to -2.29) and -3.82-point (95% CI, -6.49 to -1.14) improvement in physical functioning on a 0- to 68-point function subscale of at 3 and 6 months, respectively; and a significant improvement in patient satisfaction in 3 months with WMD -0.39 (95% CI, -0.688 to—0.36)
[41]	36	Y (21)	36	Primary outcomes: mortality, hospitalization, and clinical biomarkers or marker of disease progress. Secondary outcomes: medication adherence, economic outcomes and quality of life	RT 1- Blood pressure (OR 3.50, 95% CI 1.58 to 7.75, p = 0.002), low density lipoprotein (OR 2.35, 95% CI 1.17 to 4.72, p = 0.02), hospitalization (OR 0.69, 95% CI 0.39, 1.21, p = 0.19), mortality (OR 1.50, 95% CI 0.65 to 3.46, p = 0.34)
[42]	7	Y (5)	7	Length of hospital stay, mortality, hospital readmissions, and emergency department revisits	RT 1- Length of hospital stay (WMD = –0.04 days, 95% CI –1.63 to 1.55); mortality (OR 1.09, 95% CI 0.69 to 1.72), readmissions (OR 1.15, 95% CI 0.81 to 1.63) and emergency department revisits in 3 months (OR 0.60, 95% CI 0.27 to 1.32)
[43]	63	N (0)	63	Processes (eligibility, referral and procedure) and clinical (e.g. MRPs), humanistic (e.g. adherence) or economic outcomes (e.g. cost and effectiveness)	RT 2- Identification of MRPs (mean 3.6 MRPs per CMR) and improved adherence. Reductions in numbers of medications prescribed hospitalizations, potentially inappropriate prescribing, and costs. Qualitative research identified low awareness of CMR among eligible non-recipients, while benefits were perceived to outweigh barriers to implementation
[44]	25	Y (8)	25	Measures of health-related quality of life (HRQoL) and economic outcomes (eg. direct medical costs and incremental cost-effectiveness ratio)	RT 1—Overall, there was no significant difference in HRQoL and healthcare costs between pharmacist-provided medication review and usual care. Meta-analysis of studies that reported the 36-item Short-Form Health Survey found significant differences in favor of usual care in the body pain (mean difference: 2.94, 95% CI: 0 54–5.34, P = 0.02) and general health perception (mean difference: 1.83, 95% CI: 0.16–3.50, P = 0.03) domains, whereas there were no significant differences in other domains. Meta-analysis of the EuroQol-5D utility (mean difference: 0.01, 95% CI: 0.02–0.01, P = 0 57) and visual analogue scale (mean difference: 0.01, 95% CI: 3.24–3.26, P = 1.00) found no significant differences. Costs of hospitalization, medication, and other healthcare resources consumed were similar between groups
[45]	19	Y (16)	19	Primary: all-cause readmissions and/or emergency department visits at different time points. Secondary: all-cause readmissions, all-cause emergency department visits, drug-related readmissions, all-cause mortality, length of hospital stay, adherence, and quality of life	RT 1- All-cause readmission and/or emergency department visits (RR = 0.97, 95% CI 0.90; 1.05, p = 0.44); All-cause readmission (RR = 0.98, 95% CI 0.90; 1.06, p = 0.59); All-cause emergency department visits (RR = 0.70, 95% CI 0.59; 0.85 p = 0.0002); Drug-related readmissions (RR = 0.25, 95% CI 0.14; 0.45, p<0.0001); All-cause mortality (RR = 0.97, 95% CI 0.81; 1.17, p = 0.86); Length of hospital stay (MD -0.45 days, 95% CI -1.73; 0.82, p = 0.48)
[46]	22	N (0)	22	Medication-related outcomes (eg. medication-related problems, pharmacotherapy problems, polypharmacy, and medication appropriateness), medication review-related outcomes (eg. rate of acceptance of the recommendations by the pharmacist or team, number of recommendations, type of recommendations), and adverse outcomes (eg. potential risks such as falls, sentinel events, mortality, adverse drug events, and hospitalization)	RT 2—The majority of the recommendations put forward by the pharmacist or a multidisciplinary team was accepted by physicians. The number of prescribed medications, inappropriate medications, and adverse outcomes (eg, number of deaths, frequency of hospitalizations) were reduced in the intervention group. In the observational studies showed effective in reducing drug-related problems (DRPs), reduction in the number of medications prescribed, improvement in the medication appropriateness score, the mean number of medications per patient decreased, reduction in the number of sentinel events. Results are presented in descriptive statistics
[47]	22	N (0)	7	NR	RT 3—Reductions in the number and cost of medications prescribed; decrease in the use of antipsychotics, hypnotics, anticholinergic antidepressants, benzodiazepines, psycholeptics, psychotropics, antidepressants, potentially inappropriate medications; decrease in urinary incontinence, cognitive decline, depression scores and behavioral disorders
[48]	49	N (0)	6	Economic outcomes: cost-effectiveness and QALYs	RT 2 –Increase of cost-effectiveness and QALYs gains in the future are presented as the main economic results. In Australia, 13% of pharmacists are accredited. The main motivations for this are: professional development and satisfaction in having a more active role in patient care. Among the main barriers reported by pharmacists regarding the HMR program are: initial accreditation costs, rural locations, insufficient remuneration for the workload, lack of consumer awareness, reduced referral of patients by general practitioners, time to complete HMR (3 hours, 6 minutes)
[49]	11	Y (6)	4	Proportion of residents using one or more psychotropics	RT 3 –Reduction of the mean number of psychotropics administered per resident; a significantly greater proportion of residents ceased antipsychotic drugs and nonrecommended hypnotics; and lower proportion of residents were prescribed nonrecommended hypnotics
[50]	20	N (0)	7	Primary: use or prescribing of drugs. Secondary: health-related outcomes falls, physical limitation, hospitalization, and mortality	RT 2 –No statistically significant effect was found on drug use outcomes. No statistically significant effect was found on number of falls, patients who fell, hospitalization, and mortality. Statistically significant reduction of falls per resident
[51]	16	N (0)	3	Effect of an intervention on prescribing, aimed at improving appropriate prescribing or reducing inappropriate prescribing	RT 3—There were significant changes in the number and type of medication (medications discontinued and commenced), but the total number of medications used remained the same. There was a decline in the number of drugs prescribed with corresponding savings in drug costs, although this was not statistically different. No significant differences in drug use (total drugs and subcategories) were identified
[52]	69	N (0)	3	Medication-related falls	RT 3—Most of pharmacist’s recommendations were accepted by general practitioner. The mean number of medication changes per patient increased while the number of falls per patient decreased. There was no significant reduction in the rate of recurrent falls, injurious falls, or overall use of high-risk medications; the number of falls was reduced in the postintervention group resulting in future savings of US$7.74 per patient per day in one of the included studies. The use of the addressed drug classes decreased in the postintervention period
[53]	21	N (0)	3	QALY	RT 3—In Spain, the conSIGUE program was dominant with robust results (in terms of QALY gained, CEAC: 100%, with a WTP at 30,000€ per QALY gained), whereas in the two UK studies, the intervention was unlikely to appear cost-effective: HOMER program showed ICER = £82,678 per QALY gained, CEAC: 25%, with a WTP at £30,000 per QALY gained, only 1/5 scenarios led to an ICER< £30,000 per QALY gained while in MRpain program interventions were more costly and provide similar QALYs than usual care and results were uncertain due to the small sample size

Abbreviations: CEAC—Cost-effectiveness acceptability curve; CMR—Clinical Medication Review; CI—Confidence interval; DRPs—Drug-related problems; HRM—Home Medication Review; HRQoL—Health-related quality of life; MR—Medication Review; MRPs—Medication-related problems; OBRA—Omnibus Reconciliation Act; N—No; NDP—Numbers of drugs prescribed; OR—Odds ratio; QALY—Quality-adjusted life year; RR—Relative risk; RT 1 –Results type 1 (results from meta-analyzes of primary studies on MR); RT 2 –Results type 2 (result of the systematic review on MR); RT 3 –Results type 3 (results of the primary studies on MR); SMD—Standardized mean difference; SR—Systematic review; WMD—Weighted mean difference; WTP—Willingness to pay; Y—Yes.

Primary and secondary outcomes assessed were: mortality [37, 41, 42, 45, 50, 51], hospital visits (admissions, readmissions, hospitalizations and emergency department visits) [37, 41, 42, 45, 46, 50], drug use [37, 41, 46, 49, 51], and quality of life [37, 40, 44, 45]. Regarding the assessment of impact of MR in the meta-analyses, significant impact was not shown in any systematic review of mortality [37, 41, 42, 45], hospitalizations [37, 41], length of hospital stay [42, 45], readmission [45], readmission and/or emergency department visits [45], and revisits to emergency department [42]. MR presented significantly positive impact (p<0.05) on the all-cause emergency department visits [45], blood pressure [41], drug-related readmissions [45] intensity of pain [40], low density lipoprotein [41], number of drugs prescribed [37], quality of life [44], patients’ satisfaction [40], and physical functioning [40].

Regarding Structure variables described in the systematic reviews, the most frequent were *“pharmacists qualified to provide MR”* [39, 40, 42, 43, 44, 48], *“access to medical records”* [37–38, 40, 42], *“number of pharmacists”* [37, 39, 42, 44], and *“remuneration system”* [41, 43, 48]. Concerning the Process variables, *“number of drugs used”* [37–39, 41, 43, 46, 47, 49–52], *“number of interventions”* [38–40, 43, 46, 51], and *“accepted interventions”* [38, 40, 43, 44, 46, 52] were the most common. The most recurrent Outcomes variables, in turn, were *“mortality”* [37–39, 41–43, 45–47, 49–52], *“quality of life”* [37–45, 47, 49, 50], “*economy of costs related to drugs”* [37, 38, 41, 43, 44, 46, 47, 51], *“number of hospital visits”* [37, 41, 42, 45, 46, 50, 51], and *“patients’ satisfaction”* [37, 38, 40, 43, 47].

### Definitions, terminologies, MR approach, and interprofessional collaboration

Definitions of MR had the objective of identifying and solving DRPs and/or optimizing the drug use (Table 3) [37–39, 41–46, 48, 49]. Four systematic reviews presented components of MR, in which the most cited were: assessment of drug use history, review of patient’s medications, and health education [41, 42, 47].

**Table 3:** Definition, terminology, approach of medication review (service or intervention), and interprofessional collaboration.

Reference	Definition of MR	Main terminologies for MR	MR approach	Was there interaction between other health professionals in the MR? (What professionals?)
[37]	DP 1—Structured evaluation of a patient’s medicines, aimed at reaching agreement with the patient about drug therapy, optimizing the impact of medicines, and minimizing the number of medication related problems	*Medication Review*	Intervention	Yes (General practitioner and physician)
[38]	DP 3—To identify medication-related problems and recommending changes to optimize the medical treatment	*Clinical Medication Review*, *Medication Management Review*, *Medication Review*, and *Prescription Review*	Service	Yes (Physician)
[39]	DP 1—A structured, critical examination of a patient’s medicines with the objective of reaching an agreement with the patient about treatment, optimizing the impact of medicines, minimizing the number of medication-related problems and reducing waste	*Clinical Medication Review*, *Concordance and Compliance Review*, *Home Medicines Review*, *Medication Review*, *Prescription Review*, and *Treatment Review*	Service	Yes (General practitioner and nurse)
[40]	NR	*Medication Review*	Intervention	Yes (General practitioner, and multidisciplinary team)
[41]	DP 2—To include at least two of the following activities: reviewing patient’s medications for medication related issues; taking and documenting medication history; educating and counselling patients about medication and/or disease; providing a medication action plan; reaching an agreement with the patient about their medication treatment plan; monitoring drug treatment for effectiveness or adverse event; and optimizing medication effectiveness and minimizing problems related to medication usage	*Clinical Medication Review*, *Medication Review*, *Medicines Use Review*, *and Prescription Review*	Service	NR
[42]	DP 2—The best-possible medication history, and a review of a patient’s medications to optimize medication use, and identify and resolve medication-related problems including adverse drug events	*Medication Review*	Intervention	Yes (General practitioner, healthcare team, and physician)
[43]	DP 1—Systematic assessment of a consumer’s medications and the management of those medications, with the aim of optimizing consumer health outcomes and identifying potential medication-related issues within the framework of the quality use of medicines	*Clinical Medication Review*, *Home Medicines Review*, and *Medication Review*	Service	Yes (General practitioner, multidisciplinary team, and nurse)
[44]	DP 1—A structured, critical examination of a patient’s medicines with the objective of reaching an agreement with the patient about treatment, optimizing the impact of medicines, minimizing the number of medication-related problems and reducing waste	*Medication Review*	Intervention	Yes (Physician)
[45]	DP 1—A structured, critical examination of a patient’s medicines with the objective of reaching an agreement with the patient about treatment, optimizing the impact of medicines, minimizing the number of medication-related problems and reducing waste	*Adherence Review*, *Clinical Medication Review*, *Medication Review*, and *Prescription Review*	Intervention	Yes (Multidisciplinary team)
[46]	DP 1- A structured, critical examination of a patient’s medicines with the objective of reaching an agreement with the patient about treatment, optimizing the impact of medicines, minimizing the number of medication-related problems and reducing waste	*Clinical Medication Review*, *Concordance and Compliance Review*, *Drug Regimen Review*, *Medication Review*, *Medicines Use Review*, *Prescription Review*, and *Residential Medication Management Review*	Intervention	Yes (General practitioner, multidisciplinary team, nurse, and physician)
[47]	DP 2—Comprehensive medication history taking, patient home interviews, medication regimen review, and patient education	*Home Medicines Review*, *Medication Regimen Review*, *Medication Review*, *and Residential Medication Management Review*	Service	Yes (Multidisciplinary team, nurse, nurses’ assistant, and physician)
[48]	DP 3 –To resolve any drug-related problems to optimize drug use	*Home Medicine Review*, *Medication Review*, *and Medicines Use Review*	Service	NR
[49]	DP 1—Collaborative service provided by healthcare professionals with expertise in geriatric pharmacotherapy (usually pharmacists), designed to detect and prevent drug-related problems and hence optimize use of medicines	*Medication Review*, *Clinical Medication Review*, *Residential Medication Management Review*, and *Medication Chart Reviews*	Service	Yes (Nurses, nursing assistant, and physician)
[50]	NR	*Medication Review*	Intervention	Yes (Assistant nurse, general practitioner, multidisciplinary team, nurse, and physician)
[51]	NR	*Drug Regimen Review*, *Clinical Medication Review*, *and Medication Review*	Intervention	Yes (General practitioner, nurse, and physician)
[52]	NR	*Clinical Medication Review*, *and Medication Review*	Intervention	Yes (General practitioner, nurse, and physician)
[53]	NR	*Medication Review* and *Medicines Use Review*	Service	Yes (General practitioner)

Abbreviations: DT 1 –Definition type 1 (concept); DT 2 –Definition type 2 (components); DT 3—Definition type 3 (objectives); NR—Not reported.

The terminology *“Medication Review”* was used in all reviews (Table 3). Nine reviews used “*Clinical Medication Review*” [38, 39, 41, 45–47, 49, 51, 52] and five reviews, *“Prescription Review”* [38, 39, 41, 42, 46]. Moreover, 52.94% (n = 9) considered it as *“intervention”* [37, 40, 41, 44–46, 50–52], whilst 47.06% (n = 8) of systematic reviews considered MR as service [38, 39, 41, 43, 47–49, 53]. All reviews that used only one terminology considered MR as *“intervention”* [37, 40, 42, 43, 44].

Systematic reviews that reported interprofessional collaboration (Table 3) presented different collaborative models. Collaboration and communication occurred through direct and/or indirect contact, such as letters. The most cited health professionals were the physicians [37–40, 42, 43, 44, 46, 47, 49–52] and nurses [39, 43, 46, 47, 49–52].

### Limitations declared by systematic reviews

Main limitations described were: absence of search in grey literature [37, 39, 41, 46, 50, 53]; possibility of loss and exclusion of primary studies during search and screening processes [39, 40, 50]; number [39, 42, 45, 49] and design of included primary studies [37, 43, 45, 46]; impossibility of performing meta-analysis or limited meta-analysis due to heterogeneity of primary studies [42–44]; and restriction of language in the selection of primary studies [38, 40, 44, 46, 51, 53].

### Discussion

Most reviews had main authors and primary studies from Australia, which includes research with elderly people in community pharmacies, long-term care facilities and hospitals. Australia is one of the first countries to incorporate MR in primary outpatient care and has remuneration programs to accredited pharmacists who offer such service [54–57]. Furthermore, elderly people are some of the priority patients of this practice according to international guidelines as well as children and pregnant women [55–60]. Regarding the practice settings, Bulajeva et al. (2014) [54] corroborate our results when reported that in Europe, MR has been performed in community environments, hospitals, and long-term care facilities.

Terminologies for MR are not standardized in literature. Among the most well-known are: *Home Medicines Review*, *Medication Use Review* and *Residential Medication Management Review*, in Australia [61–63]; *Revisión de la medicación* and *Revisión sistemática de la medicación*, in Spain [64, 65]; *Medication Review and Comprehensive Medication Review*, in the United States [66, 67]; *Comprehensive Medication Review*, in Finland [68, 69]; and *Medicines Use Review* in the United Kingdom [70, 71]. These terminologies result from the differences in patient complexity and characteristics of each country and practice setting where MR is performed [72–74].

In pharmacy, there is no consensus among concepts and terminologies of clinical practice [75–79]. Linguistic and cultural questions as well as the overlapping between *“what we do”* and *“how we do”* can be causes of these divergences. Consequently, lack of standardization of definitions and terminologies can confuse researchers and professionals who aim to compare results and to confirm the practice effectiveness [75, 78]. Thus, definitions and terminologies internationally standardized can benefit the assessment of impact of MR [80]. Moreover, modelling of clinical pharmacy services should be used since it facilitates the standardization and comparison of MR and provides a holistic approach to the decision-making process and organizational change [81–85]. Therefore, establishing minimum quality standards for MR is important for comparison of the practice as well as for the optimization of the care provided and, consequently, of the patients’ health outcomes.

Regardless of the terminology adopted in systematic reviews, the objective of MR, both as service as well as intervention, is to identify and solve DRPs, implement changes in patients’ pharmacotherapy and improve health outcomes. This objective agreed with *guidelines* of the countries where this practice is more frequent. In this regard, interprofessional collaboration is necessary to reach this objective [55–60, 86, 87]. Collaborations among healthcare professionals declared in the systematic reviews can be seen as a positive factor to achieve better clinical, economic, and humanistic outcomes.

Interprofessional collaboration can be encouraged through specializations, since they motivate information sharing and communication between healthcare professionals. Furthermore, appropriate training of these professionals, such as pharmacists, is essential to develop abilities to the clinical practice, for example, critical thinking and collaborative interpersonal practice [88]. In Australia, only pharmacists who are trained and go through assessments are accredited and can provide MR [89]. In the United States, post-graduate pharmacist residency training, as well as physician residency training, has become a requirement for entry-level health-system pharmacy practice [90]. In this same country, the Accreditation Council for Pharmacy Education (ACPE) (2016) [91] established interprofessional collaboration as one of the accreditation standards for the professional program in Pharmacy leading to the Doctor of Pharmacy degree. According to this institution, the curriculum should prepare students to provide entry-level, patient-centered care in different practice settings as member of an interprofessional team (with prescribers or other healthcare professional). Thus, literature supports our finding of *"pharmacists qualified to provide MR"* as a frequently described structure variable.

In this context, MR can be performed by physicians, nurses, and pharmacists. Despite using the same terminology, for physicians and nurses, MR is usually a component of clinical practice whose process has not been well described in literature [92, 93]. In pharmacy, studies and guidelines of different countries described MR as a clinical pharmacy service or intervention [13, 16, 94–100]. In this overview, the systematic reviews who presented practice components of MR (e.g. assessment of drug use history, health education, and review of patient’s medications) addressed it as service or intervention. However, the discrepancy between the MR approach as an intervention and the concept of the intervention present in the literature is noticeable.

According to the Society of Hospital Pharmacists of Australia, *“intervention”* is *“any action performed by a clinical pharmacist that directly results in the change of patient management or therapy”* [101]. Suggett and Marriott (2016) [102], in turn, define *“intervention”* as a process in which the pharmacist identifies and makes recommendations in an attempt to prevent or resolve DRPs. The authors emphasize that the definition of “*intervention*” does not include MR without recommendations for changes in treatment.

In Brazil, *“intervention”* is a *“professional action planned*, *documented and performed by the pharmacist for optimization of pharmacotherapy*, *promotion*, *protection and recovery of health*, *prevention of diseases and other health problems”* [103]. Thus, we understand *"pharmacist intervention"* as an action whose goal is to improve patient health outcomes and that may result in changes in pharmacotherapy. In addition, *"intervention"* is a result of the situational analysis of the patient, and is part of the care plan, step of the patient care process.

Considering the patient care process, three stages are recommended in several health professions, including pharmacists: initial assessment, care plan, and assessment of outcomes. The first stage is a situational analysis in which the pharmacist gathers, analyzes and interprets information about patient’s clinical conditions and pharmacotherapy, aiming to evaluate his or her drug-related needs. The second stage is the care plan whose purpose is to agree with the patient the actions necessary to manage his or her clinical conditions successfully with pharmacotherapy. The care plan includes goals of therapy, interventions (e.g. inclusion of new drug therapy, patient education, and referrals to other health professionals), and a schedule for assessment of outcomes. In the third stage, patient outcomes are assessed, documented, and compared to the goals of therapy [104].

From the presented patient care process, the most appropriate MR approaches are as *"service"* or *"practice component"*. As a service, MR should include all steps of the patient care process. As a practice component, MR is part of other health services, such as medication reconciliation, and consists of the situational analysis of the patient’s pharmacotherapy. Hence, future studies need to describe if MR is a clinical pharmacy service or a practice component. Only then, it will be possible to compare the impact of its results and assure the robustness of this practice.

Regardless of the MR approach, access to medical records is important for the clinical practice. Literature has reported that pharmacists should rely on medical records and technical drug information to make decisions based on evidences and provides the best possible patient care [105, 106]. Guidelines highlight the need to obtain patient information from different sources, such as interviews with patient and caregiver, clinical laboratory tests, and medical records, considering that they are complementary [55–60]. Therefore, describing access to medical records as a structure variable is relevant, since the limitation of access to any sources of information can result in the restriction of clinical activities of pharmacists.

Although there is no standardization for terminologies and approaches of MR, there are variables related to the care process that are commonly used in this practice, such as: *“number of drugs”* and *“number of interventions”*. Considering the objective of MR, literature confirms that the analysis of the number of drugs is necessary to the assessment of the impact of MR, especially because it can involve vulnerable patients which present polypharmacy, therapeutic duplication, drug interactions, and contraindications [55–60]. Thus, Cipolle et al. (2012) highlight that interventions related to the resolution of DRPs, usually in interprofessional collaboration, may result in the reduction of the number of drugs [104].

Despite Jokanovic et al. (2016) [27] mentioning the positive impact of MR in primary studies performed in community environments (e.g. blood pressure control, quality of life, and healthcare costs), some reviews included in our study show that MR results were contradictory, had little significant or were inconclusive. A systematic review conducted by Huiskes et al. (2017) [107] showed positive and negative effects for some outcomes variables described in our overview. According to the authors, the different results found may be a reflection of factors such as: 1) selection of patients, which may not fit the objective of MR; 2) how MR is performed in the clinical practice, since there is heterogeneity in the work processes and there is no gold standard on how it should be performed; and 3) outcomes and time of follow-up used to assess the impact of MR, variables that should be chosen according to the objective of MR and being more specific to diseases and drugs. Thus, heterogeneity of processes can affect the method of data analysis as well as the sensitivity and specificity of results, such as mortality, economy of drugs costs, hospital readmissions, quality of life and patients’ satisfaction.

Another factor that influences the impact analysis of MR is the methodological quality of the reviews. Although systematic reviews are considered a key element used to the practices of patient care, low quality of reviews have limited processes of decision making and performance of healthcare systems. Our findings were corroborated by literature that has reported low methodological quality of systematic reviews on clinical pharmacy practice. Melchiors et al. (2012) [108] assessed the quality of 31 systematic reviews, in which 24 presented low and moderate quality. In overview of seven systematic reviews, Aguiar et al. (2014) [109] noticed that 71.4% of the reviews had low and moderate quality. Rotta et al. (2015) [110], in turn, found in overview of 49 systematic reviews that no review met all AMSTAR criteria. Therefore, future systematic reviews should value high methodological quality to result in more reliable evidences of real impact of clinical services.

### Strengths and limitations

Strengths of this overview include: research in six different databases as well as manual search in the references of the included systematic reviews; use of MESH terms and text words for the literature search; use of 15 different terminologies to MR in the literature search; title, abstract, and full text screenings as well as quality assessment performed by two independent investigators. Moreover, systematic reviews were not excluded based on methodological quality, study design, practice setting, and population. Thus, our study presents a panorama of systematic reviews about pharmacist-participated MR. This overview comprised variables little explored in overviews of systematic reviews on MR, such as definitions and terminologies of MR; interprofessional collaboration; MR approach as well as structure, processes, and outcomes variables described in the systematic reviews.

This overview also presents limitations. Search in the grey literature was not performed. As most systematic reviews are found in databases, the inclusion of only indexed reviews may not have influenced the final sample. Data extraction and analysis of the variables studied were based on systematic reviews rather than primary studies, which may have resulted in overlapping of primary studies in the evaluation of results of MR. Many included reviews did not provide clear information or presented few details on primary studies’ design, population, and practice setting; definitions of MR; and impact of MR, this might have compromised data extraction. Moreover, AMSTAR limitations, such as the subjectivity of items *“no”* and *“cannot answer”* and the dependence of quality of reports [111, 112], could have influenced the assessment of methodological quality of systematic reviews included in this overview.

## Toward a future research agenda

This overview of systematic reviews is a starting point to analyze the panorama of literature on pharmacist-participated MR in different practice settings regarding to the concepts, terminologies and approach of MR as well as interprofessional collaboration. From the findings of this overview, it is possible to identify the need for future systematic reviews and primary studies to clarify these variables. The lack of clarity about concepts, terminologies and MR approach as well as interprofessional collaboration extracted from the primary studies of the included systematic reviews may be due to the summarization of the results found in these primary studies and/or to the lack of clarity of the primary studies themselves. Thus, future systematic reviews should analyze these variables in the primary studies in order to reinforce the need to standardize concepts, terminologies and approach of MR in the literature. Moreover, the findings of this overview should also be addressed in future primary studies since any systematic review is only as good as the primary studies that compose it. That is, having in the literature high quality of systematic reviews is just as important as having high quality primary studies. Therefore, future studies, both systematic reviews and primary studies, should clearly present the variables studied in this manuscript in order to facilitate the understanding of effectiveness of MR and the comparison of its results.

## Conclusion

In this overview, considerable heterogeneity of systematic reviews about MR was evidenced, especially regarding practice setting, population, MR approach and terminology. Description of patient care process of the primary studies is not clear in some reviews. These facts may limit the comparison, summarization and understanding of MR results. *“Medication Review”* was the most used terminology, whose main objective is the identification and resolution of DRPs to optimize the drug use. MR practice is mostly comprehended as *“intervention”*, and its main collaborator is the physician. Moreover, methodological quality of most systematic reviews was below ideal. In the light of what was mentioned, it is necessary to come to an international agreement regarding the work process of MR, as a clinical service or practice component, improving, then, the assessment, comparison and optimization of care quality given to patients.

## Supporting information

S1 Table Search strategy in databases. (DOC)

S2 Table List of excluded studies and reasons for exclusion. Abbreviation: MR—Medication Review.
(DOC)

S1 Checklist PRISMA checklist. Abbreviations: NA: Not Applicable; U: Unrealized.
(DOC)
