# Supplementary material for: Pharmacist-participated medication review in different practice settings: Service or intervention? An overview of systematic reviews
Source: PLoS One. 2019 Jan 10;14(1):e0210312. doi: 10.1371/journal.pone.0210312 (PMC6328162; doi:10.1371/journal.pone.0210312)
Supplement: S1 Table — (DOC) [file pone.0210312.s001.doc]

**S1 Table - Search strategy in databases**

| *Bliblioteca Virtual em Saúde (BVS)* | #1 | (tw:((mh:"drug utilization review") OR "drug utilization review" OR "evaluation, drug utilization" OR "drug-use review" OR "drug use review" OR "drug-use reviews" OR "reviews, drug-use" OR "review, drug utilization" OR "drug utilization reviews" OR "reviews, drug utilization" OR "utilization reviews, drug" OR "utilization review, drug" OR "drug utilization evaluation" OR "drug utilization evaluations" OR "evaluations, drug utilization" OR "utilization evaluation, drug" OR "utilization evaluations, drug" OR "review, drug-use" OR "review, drug use" OR "medication review" OR "medication reviews" OR "home medicines review" OR "home medicines reviews" OR "medicines use review" OR "medicines use reviews" OR "medication use review" OR "medication use reviews" OR "residential medication management review" OR "residential medication management reviews" OR "prescription review" OR "prescription reviews" OR "treatment review" OR "treatment reviews" OR "clinical medication review" OR "clinical medication reviews" OR "concordance and compliance review" OR "concordance and compliance reviews" OR "medication chart review" OR "medication chart reviews" OR "medication therapy review" OR "medication therapy reviews" OR "medication regimen review" OR "medication regimen reviews" OR "brown bag review" OR "brown bag reviews")) AND (tw:((mh:"pharmacists") OR "pharmacists" OR "pharmacist" OR "clinical pharmacists" OR "clinical pharmacist" OR "pharmacist, clinical" OR "pharmacists, clinical" )) AND (tw:("systematic review" OR "systematic reviews" OR "meta-analysis" OR "systematic literature review" OR "systematic literature reviews")) |
| --- | --- | --- |
| *Embase* | #1 | 'drug utilization review'/exp OR 'drug utilization review' OR 'evaluation, drug utilization' OR 'drug-use review'/exp OR 'drug-use review' OR 'drug use review'/exp OR 'drug use review' OR 'drug-use reviews' OR 'reviews, drug-use' OR 'review, drug utilization' OR 'drug utilization reviews' OR 'reviews, drug utilization' OR 'utilization reviews, drug' OR 'utilization review, drug' OR 'drug utilization evaluation'/exp OR 'drug utilization evaluation' OR 'drug utilization evaluations' OR 'evaluations, drug utilization' OR 'utilization evaluation, drug' OR 'utilization evaluations, drug' OR 'review, drug-use' OR 'review, drug use' OR 'medication review'/exp OR 'medication review' OR 'medication reviews' OR 'home medicines review' OR 'home medicines reviews' OR 'medicines use review' OR 'medicines use reviews' OR 'medication use review'/exp OR 'medication use review' OR 'medication use reviews' OR 'residential medication management review' OR 'residential medication management reviews' OR 'prescription review' OR 'prescription reviews' OR 'treatment review' OR 'treatment reviews' OR 'clinical medication review' OR 'clinical medication reviews' OR 'concordance and compliance review' OR 'concordance and compliance reviews' OR 'medication chart review' OR 'medication chart reviews' OR 'medication therapy review' OR 'medication therapy reviews' OR 'medication regimen review' OR 'medication regimen reviews' OR 'brown bag review' OR 'brown bag reviews' |
|  | #2 | 'pharmacists'/exp OR 'pharmacists' OR 'pharmacist'/exp OR 'pharmacist' OR 'clinical pharmacists' OR 'clinical pharmacist'/exp OR 'clinical pharmacist' OR 'pharmacist, clinical' OR 'pharmacists, clinical' |
|  | #3 | 'systematic review'/exp OR 'systematic review' OR 'systematic reviews'/exp OR 'systematic reviews' OR 'meta-analysis'/exp OR 'meta-analysis' OR 'systematic literature review' OR 'systematic literature reviews' |
|  | #4 | #1 AND # 2 AND #3 |
| *PubMed* | #1 | “drug utilization review” [MeSH Terms]) OR (“drug utilization review” or “evaluation, drug utilization” or “drug-use review” or “drug use review” or “drug-use reviews” or “reviews, drug-use” or “review, drug utilization” or “drug utilization reviews” or “reviews, drug utilization” or “utilization reviews, drug” or “utilization review, drug” or “drug utilization evaluation” or “drug utilization evaluations” or “evaluations, drug utilization” or “utilization evaluation, drug” or “utilization evaluations, drug” or “review, drug-use” or “review, drug use” or “medication review” or “medication reviews” or “home medicines review” or “home medicines reviews” or “medicines use review” or “medicines use reviews” or “medication use review” or “medication use reviews” or “residential medication management review” or “residential medication management reviews” or “prescription review” or “prescription reviews” or “treatment review” or “treatment reviews” or “clinical medication review” or “clinical medication reviews” or “concordance and compliance review” or “concordance and compliance reviews” or “medication chart review” or “medication chart reviews” or “medication therapy review” or “medication therapy reviews” or “medication regimen review” or “medication regimen reviews” or “brown bag review” or “brown bag reviews” |
|  | #2 | “pharmacists” [MeSH Terms]) OR (“pharmacists” or “pharmacist” or “clinical pharmacists” or “clinical pharmacist” or “pharmacist, clinical” or “pharmacists, clinical” |
|  | #3 | “systematic review” or “systematic reviews” or “meta-analysis” or “systematic literature review” or “systematic literature reviews” |
|  | #4 | #1 AND # 2 AND #3 |
| *Scopus* | #1 | "drug utilization review" OR "evaluation, drug utilization" OR "drug-use review" OR "drug use review" OR "drug-use reviews" OR "reviews, drug-use" OR "review, drug utilization" OR "drug utilization reviews" OR "reviews, drug utilization" OR "utilization reviews, drug" OR "utilization review, drug" OR "drug utilization evaluation" OR "drug utilization evaluations" OR "evaluations, drug utilization" OR "utilization evaluation, drug" OR "utilization evaluations, drug" OR "review, drug-use" OR "review, drug use" OR "medication review" OR "medication reviews" OR "home medicines review" OR "home medicines reviews" OR "medicines use review" OR "medicines use reviews" OR "medication use review" OR "medication use reviews" OR "residential medication management review" OR "residential medication management reviews" OR "prescription review" OR "prescription reviews" OR "treatment review" OR "treatment reviews" OR "clinical medication review" OR "clinical medication reviews" OR "concordance and compliance review" OR "concordance and compliance reviews" OR "medication chart review" OR "medication chart reviews" OR "medication therapy review" OR "medication therapy reviews" OR "medication regimen review" OR "medication regimen reviews" OR "brown bag review" OR "brown bag reviews" |
|  | #2 | "pharmacists" OR "pharmacist" OR "clinical pharmacists" OR "clinical pharmacist" OR "pharmacist, clinical" OR "pharmacists, clinical" |
|  | #3 | "systematic review" OR "systematic reviews" OR "meta-analysis" OR "systematic literature review" OR "systematic literature reviews" |
|  | #4 | #1 AND #2 AND #3 |
| *The Cochrane Library* | #1 | MeSH descriptor: [Drug Utilization Review] explode all trees |
|  | #2 | "drug utilization review" or "evaluation, drug utilization" or "drug-use review" or "drug use review" or "drug-use reviews" or "reviews, drug-use" or "review, drug utilization" or "drug utilization reviews" or "reviews, drug utilization" or "utilization reviews, drug" or "utilization review, drug" or "drug utilization evaluation" or "drug utilization evaluations" or "evaluations, drug utilization" or "utilization evaluation, drug" or "utilization evaluations, drug" or "review, drug-use" or "review, drug use" or "medication review" or "medication reviews" or "home medicines review" or "home medicines reviews" or "medicines use review" or "medicines use reviews" or "medication use review" or "medication use reviews" or "residential medication management review" or "residential medication management reviews" or "prescription review" or "prescription reviews" or "treatment review" or "treatment reviews" or "clinical medication review" or "clinical medication reviews" or "concordance and compliance review" or "concordance and compliance reviews" or "medication chart review" or "medication chart reviews" or "medication therapy review" or "medication therapy reviews" or "medication regimen review" or "medication regimen reviews" or "brown bag review" or "brown bag reviews" |
|  | #3 | #1 or #2 |
|  | #4 | MeSH descriptor: [Pharmacists] explode all trees |
|  | #5 | "pharmacists" or "pharmacist" or "clinical pharmacists" or "clinical pharmacist" or "pharmacist, clinical" or "pharmacists, clinical" |
|  | #6 | #4 or #5 |
|  | #7 | "systematic review" or "systematic reviews" or "meta-analysis" or "systematic literature review" or "systematic literature reviews" |
|  | #8 | #3 and #6 and #7 |
| *Web of Science* | #1 | Tópico: (“drug utilization review” or “evaluation, drug utilization” or “drug-use review” or “drug use review” or “drug-use reviews” or “reviews, drug-use” or “review, drug utilization” or “drug utilization reviews” or “reviews, drug utilization” or “utilization reviews, drug” or “utilization review, drug” or “drug utilization evaluation” or “drug utilization evaluations” or “evaluations, drug utilization” or “utilization evaluation, drug” or “utilization evaluations, drug” or “review, drug-use” or “review, drug use” or “medication review” or “medication reviews” or “home medicines review” or “home medicines reviews” or “medicines use review” or “medicines use reviews” or “medication use review” or “medication use reviews” or “residential medication management review” or “residential medication management reviews” or “prescription review” or “prescription reviews” or “treatment review” or “treatment reviews” or “clinical medication review” or “clinical medication reviews” or “concordance and compliance review” or “concordance and compliance reviews” or “medication chart review” or “medication chart reviews” or “medication therapy review” or “medication therapy reviews” or “medication regimen review” or “medication regimen reviews” or “brown bag review” or “brown bag reviews”)  Índices=SCI-EXPANDED, SSCI, A&HCI, CPCI-S, CPCI-SSH, ESCI Tempo estipulado=Todos os anos |
|  | #2 | Tópico: (“pharmacists” or “pharmacist” or “clinical pharmacists” or “clinical pharmacist” or “pharmacist, clinical” or “pharmacists, clinical”)  Índices=SCI-EXPANDED, SSCI, A&HCI, CPCI-S, CPCI-SSH, ESCI Tempo estipulado=Todos os anos |
|  | #3 | Tópico: (“systematic review” or “systematic reviews” or “meta-analysis” or “systematic literature review” or “systematic literature reviews”)  Índices=SCI-EXPANDED, SSCI, A&HCI, CPCI-S, CPCI-SSH, ESCI Tempo estipulado=Todos os anos |
|  | #4 | #3 AND #2 AND #1  Índices=SCI-EXPANDED, SSCI, A&HCI, CPCI-S, CPCI-SSH, ESCI Tempo estipulado=Todos os anos |
