# Supplementary material for: Pharmacist-participated medication review in different practice settings: Service or intervention? An overview of systematic reviews
Source: PLoS One. 2019 Jan 10;14(1):e0210312. doi: 10.1371/journal.pone.0210312 (PMC6328162; doi:10.1371/journal.pone.0210312)
Supplement: S2 Table — Abbreviation: MR—Medication Review. (DOC) [file pone.0210312.s002.doc]

**S2 Table – List of excluded studies and reasons for exclusion**

| **Authors, year** | **Title** | **Reference** | **Reason for exclusion** |
| --- | --- | --- | --- |
| Richardson et al. (2014) | A comprehensive review of the impact of clinical pharmacy services on patient outcomes in mental health | Int J Clin Pharm.; 36(2):222-32 | Results did not separate MR from other services / interventions |
| Nordin et al. (2017) | A global picture of extended pharmacy services, perceptions, and barriers toward its performance: a systematic review | Asian J Pharm Clin Res.; 10(11):417-27 | Results did not separate MR from other services / interventions |
| Michelazzo et al. (2017) Michelazzo | A systematic review of case-series studies on the effectiveness of interventions to reduce polypharmacy and its adverse consequences in the elderly | Epidemiol Biostat Public Health.; 14(1): e12148-1 | MR was not evaluated |
| George et al. (2008) | A systematic review of interventions to improve medication taking in elderly patients prescribed multiple medications | Drugs Aging.; 25(4):307-24. | Results did not separate MR from other services / interventions |
| Beuscart et al. (2017) | A systematic review of the outcomes reported in trials of medication review in older patients: the need for a core outcome set | Br J Clin Pharmacol.; 83(5):942-52 | MR was not led by a pharmacist in all primary studies |
| Pickard and Hung (2006) | An update on evidence of clinical pharmacy services' impact on health-related quality of life | Ann Pharmacother.; 40(9):1623-34. | Results did not separate MR from other services / interventions |
| Da Silva et al. (2010) | Analysis of research quality regarding pharmaceutical intervention in elderly residents of long-term care facilities: a systematic review | Am Soc Geriatr; 58(7): 1404-6 | Results did not separate MR from other services / interventions |
| Raae-Hansen et al. (2017) | Changing behaviours: A systematic literature review of deprescribing interventions in older people | Age Ageing.; 46(3):iii13–iii59 | Not a systematic review (conference paper) |
| Tan et al. (2016) | Clinical and economic outcomes of pharmacist-provided home-based medication review in the elderly: a systematic review and meta-analysis | Value Health.; 19(7): A907 | Not a systematic review (conference paper) |
| Tanna et al. (2015) | Clinical medication review as part of an elderly fallers patient care screening service | Maturitas 81(1):221-22 | Not a systematic review (conference paper) |
| Kaboli et al. (2006) | Clinical pharmacists and inpatient medical care: a systematic review | Arch Intern Med.; 166(9):955-64 | Results did not separate MR from other services / interventions |
| Sadowski et al. (2010) | Contributions of home-based medication reviews in the elderly: a systematic review | J Am Geriatr Soc; 58: S52-S53 | Not a systematic review (conference paper) |
| Akazawa (2017) | Cost-effectiveness analysis of medication review interventions: a review of literatures | Value Health.;20(9): A515 | Not a systematic review (conference paper) |
| Malet-Larrea et al. (2016) | Cost-effectiveness of professional pharmacy services in community pharmacy: a systematic review | Expert Rev Pharmacoecon Outcomes Res.; 16(6):747-58 | Results did not separate MR from other services / interventions |
| Gammie et al. (2017) | Economic evaluation of hospital and community pharmacy services | Ann Pharmacother.; 51(1):54-65 | Results did not separate MR from other services / interventions |
| Wang et al. (2016) | Economic evaluations of pharmacist-managed services in people with diabetes mellitus: a systematic review | Diabet Med.; 33(4):421-7 | Results did not separate MR from other services / interventions |
| Blalock et al. (2013) | Economic impact of pharmacist services in the community setting | Med Care Res Rev.; 70(3): 235–266 | Not a systematic review (conference paper) |
| Cohen et al. (2005) | Effect of clinical pharmacists on care in the emergency department: a systematic review | Br J Gen Pract.; 55(520): 875–882 | MR was not evaluated |
| Nkansah et al. (2010) | Effect of outpatient pharmacists' non-dispensing roles on patient outcomes and prescribing patterns | Cochrane Database Syst Rev.; (7):CD000336 | Results did not separate MR from other services / interventions |
| El Hajj et al. (2017) | Effect of pharmacist care on medication adherence and cardiovascular outcomes among patients post-acute coronary syndrome: a systematic review | Res Social Adm Pharm. pii: S1551-7411(17)30370-4 | Not a systematic review (conference paper) |
| Readdean et al. (2018) | Effect of pharmacist intervention on improving antidepressant medication adherence and depression symptomology: a systematic review and meta-analysis | Res Social Adm Pharm.;14(4):321-31 | MR was not evaluated |
| Neyens et al. (2011) | Effectiveness and implementation aspects of interventions for preventing falls in elderly people in long-term care facilities: a systematic review of RCTs | J Am Med Dir Assoc.; 12(6):410-25 | Results did not separate MR from other services / interventions |
| Van Wijk et al. (2005) | Effectiveness of interventions by community pharmacists to improve patient adherence to chronic medication: a systematic review | Ann Pharmacother.; 39(2):319-28 | Results did not separate MR from other services / interventions |
| Huiskes et al. (2017) | Effectiveness of medication review: a systematic review and meta-analysis of randomized controlled trials | BMC Fam Pract. 2017; 18: 5 | MR was not led by a pharmacist in all primary studies |
| Rubio-Valera et al. (2011) | Effectiveness of pharmacist care in the improvement of adherence to antidepressants: a systematic review and meta-analysis | Ann Pharmacother.; 45(1):39-48 | MR was not evaluated |
| Mansah et al. (2009) | Effectiveness of strategies to promote safe transition of elderly people across care settings | JBI Libr Syst Rev.; 7(24):1036-90 | Results did not separate MR from other services / interventions |
| Zhong et al. (2014) | Evaluation of pharmacist care for patients with chronic obstructive pulmonary disease: a systematic review and meta-analysis | Int J Clin Pharm.; 36(6):1230-40 | Results did not separate MR from other services / interventions |
| Sáez-Benito et al. (2013) | Evidence of the clinical effectiveness of cognitive pharmaceutical services for aged patients | Age Ageing.; 42(4):442-9 | Not a systematic review |
| Lang et al. (2014) | How can we reduce inappropriate prescribing of antipsychotic medications for people with dementia in residential care settings? a systematic review of implementation strategies | Alzheimers Dement.; 10(4):P737 | Not a systematic review (conference paper) |
| Loh et al. (2016) | Humanistic and economic outcomes of pharmacist-provided medication reviews in the elderly: a systematic review and meta-analysis | Value Health 19(7):A814 | Not a systematic review (conference paper) |
| Ensing et al. (2015) | Identifying the optimal role for pharmacists in care transitions: a systematic review | J Manag Care Spec Pharm.; 21(8):614-36. | Results did not separate MR from other services / interventions |
| Santschi et al. (2011) | Impact of pharmacist care in the management of cardiovascular disease risk factors: a systematic review and meta-analysis of randomized trials | Arch Intern Med.; 171(16):1441-53 | MR was not evaluated |
| Al-Jumah and Qureshi (2012) | Impact of pharmacist interventions on patients' adherence to antidepressants and patient-reported outcomes: a systematic review | Patient Prefer Adherence.; 6:87-100 | MR was not evaluated |
| Tully and Seston, (2000) | Impact of pharmacists providing a prescription review and monitoring service in ambulatory care or community practice | Ann Pharmacother.; 34(11):1320-31 | Results did not separate MR from other services / interventions |
| Santschi et al. (2014) | Improving blood pressure control through pharmacist interventions: a meta-analysis of randomized controlled trials | J Am Heart Assoc.; 3:e000718 | MR was not evaluated |
| Kucukarslan et al. (2011) | Integrating medication therapy management in the primary care medical home: A review of randomized controlled trials | Am J Health Syst Pharm.; 68(4):335-45 | Focused on other pharmaceutical service |
| Clegg et al. (2014) | Interventions for preventing delirium in older people in institutional long-term care | Cochrane Database Syst Rev.; (1):CD009537 | Results did not separate MR from other services / interventions |
| Royal et al. (2006) | Interventions in primary care to reduce medication related adverse events and hospital admissions: systematic review and meta-analysis | Qual Saf Health Care.; 15(1):23-31. | Results did not separate MR from other services / interventions |
| McGrattan et al. (2017) | Interventions to improve medicines management for people with dementia: a systematic review | Drugs Aging.; 34(12):907-16 | Full-text not available |
| Alldred et al. (2016) | Interventions to optimise prescribing for older people in care homes | Cochrane Database Syst Rev.; 2:CD009095 | Results did not separate MR from other services / interventions |
| Gutiérrez Valencia et al. (2016) | Interventions to optimize pharmacologic treatment in hospitalized older adults: a systematic review | Rev Clin Esp.;216(4):205-21 | Results did not separate MR from other services / interventions |
| Thompson et al. (2014) | Interventions to reduce inappropriate prescribing of antipsychotic medications in people with dementia resident in care homes: a systematic review | J Am Med Dir Assoc.; 15(10):706-18 | MR was not led by a pharmacist in all primary studies |
| Manias et al. (2014) | Interventions to reduce medication errors in pediatric intensive care | Ann Pharmacother; 48(10):1313-31. | MR was not evaluated |
| Fahey et al. (2006) | Interventions used to improve control of blood pressure in patients with hypertension | Cochrane Database Syst Rev.; 19(2):CD005182. | MR was not evaluated |
| Glynn et al. (2010) | Interventions used to improve control of blood pressure in patients with hypertension | Cochrane Database Syst Rev. 17;(3):CD005182 | MR was not evaluated |
| Aguiar et al., (2016) | Investigating sources of heterogeneity in randomized controlled trials of the effects of pharmacist interventions on glycemic control in type 2 diabetic patients: a systematic review and meta-analysis | PLoS One.; 11(3):e0150999 | Results did not separate MR from other services / interventions |
| Geurts et al. (2012) | Medication review and reconciliation with cooperation between pharmacist and general practitioner and the benefit for the patient: a systematic review | Br J Clin Pharmacol.; 74(1):16-33 | Results did not separate MR from other services / interventions |
| Christensen and Lundh, (2016) | Medication review in hospitalised patients to reduce morbidity and mortality | Cochrane Database Syst Rev.; 20:CD008986 | MR was not led by a pharmacist in all primary studies |
| Aston et al. (2016) | Medication review of children on long term medications: a review of the literature | Arch Dis Child.; 101(9):e2 | Not a systematic review (conference paper) |
| Wallerstedt et al. (2014) | Medication reviews for nursing home residents to reduce mortality and hospitalization: systematic review and meta-analysis | Br J Clin Pharmacol.; 78(3): 488–497 | MR was not led by a pharmacist in all primary studies |
| Viswanathan et al. (2015) | Medication therapy management interventions in outpatient settings: a systematic review and meta-analysis | JAMA Intern Med.; 175(1):76-87 | Focused on other pharmaceutical service |
| Asayut et al. (2018) | Outcomes, costs and stakeholders' perspectives associated with the incorporation of community pharmacy services into the National Health Insurance System in Thailand: a systematic review | Int J Pharm Pract.;26(1):16-27 | MR was not evaluated |
| Willeboordse et al. (2014) | Patient participation in medication reviews is desirable but not evidence-based: a systematic literature review | Br J Clin Pharmacol.; 78(6):1201-16 | MR was not led by a pharmacist in all primary studies |
| Houle et al. (2014) | Paying pharmacists for patient care: a systematic review of remunerated pharmacy clinical care services | Can Pharm J (Ott).; 147(4):209-32 | Results did not separate MR from other services / interventions |
| Aguiar et al. (2012) | Pharmaceutical care in hypertensive patients: a systematic literature review | Res Social Adm Pharm.; 8(5):383-96 | MR was not evaluated |
| Roughead et al. (2005) | Pharmaceutical care services: a systematic review of published studies, 1990 to 2003, examining effectiveness in improving patient outcomes | Int J Pharm Pract.; 13(1): 53-70 | Results did not separate MR from other services / interventions |
| Cai et al. (2013) | Pharmacist care and the management of coronary heart disease: a systematic review of randomized controlled trials | BMC Health Serv Res; 13:461 | Results did not separate MR from other services / interventions |
| Morgado et al. (2011) | Pharmacist interventions to enhance blood pressure control and adherence to antihypertensive therapy: review and meta-analysis | Am J Health Syst Pharm.; 68(3):241-53 | MR was not evaluated |
| Santschi et al. (2012) | Pharmacist interventions to improve cardiovascular disease risk factors in diabetes: a systematic review and meta-analysis of randomized controlled trials | Diabetes Care.; 35(12):2706-17 | Results did not separate MR from other services / interventions |
| Tan et al. (2014) | Pharmacist services provided in general practice clinics: a systematic review and meta-analysis | Res Social Adm Pharm.; 10(4):608-22 | Results did not separate MR from other services / interventions |
| Kang et al. (2016) | Pharmacist-involved care for patients with heart failure and acute coronary syndrome: a systematic review with qualitative and quantitative meta-analysis | [J Clin Pharm Ther.](http://www.ncbi.nlm.nih.gov/pubmed/26954666) ; 41(2):145-57 | MR was not evaluated |
| Thomas et al. (2014) | Pharmacist-led interventions to reduce unplanned admissions for older people: a systematic review and meta-analysis of randomised controlled trials | [Age Ageing.](http://www.ncbi.nlm.nih.gov/pubmed/24196278); 43(2):174-87 | Results did not separate MR from other services / interventions |
| Basaraba et al. (2018) | Pharmacists as care providers for stroke patients: a systematic review | Can J Neurol Sci.; 45(1):49-55 | Results did not separate MR from other services / interventions |
| Verrue et al. (2009) | Pharmacists' interventions for optimization of medication use in nursing homes: a systematic review | [Drugs Aging.](http://www.ncbi.nlm.nih.gov/pubmed/19102513); 26(1):37-49 | Results did not separate MR from other services / interventions |
| Salgado et al. (2012) | Pharmacists' interventions in the management of patients with chronic kidney disease: a systematic review | Nephrol Dial Transplant.; 27(1):276-92 | Results did not separate MR from other services / interventions |
| Garcia-Cardenas et al. (2016) | Pharmacists' interventions on clinical asthma outcomes: a systematic review | Eur Respir J.; 47(4):1134-43 | MR was not evaluated |
| O'Dwyer et al. (2015) | Pharmacists' medicines-related interventions for people with intellectual disabilities: a narrative review | Int J Clin Pharm.;37(4):566-78 | Results did not separate MR from other services / interventions |
| Mason (2011) | Polypharmacy and medication-related complications in the chronic kidney disease patient | Curr Opin Nephrol Hypertens.;20(5):492-7 | Not a systematic review |
| Fish et al. (2002) | Practice-based pharmaceutical services: a systematic review | Int J Pharm Pract.; 10(4): 225-33 | Results did not separate MR from other services / interventions |
| Machado et al. (2007) | Sensitivity of patient outcomes to pharmacist interventions. Part II: Systematic review and meta-analysis in hypertension management | Ann Pharmacother.; 41(11):1770-81 | MR was not evaluated |
| Machado et al. (2008) | Sensitivity of patient outcomes to pharmacist interventions. Part III: systematic review and meta-analysis in hyperlipidemia management | Ann Pharmacother.; 42(9):1195-207 | MR was not evaluated |
| Machado et al. (2007) | Sensitivity of patient outcomes to pharmacist interventions. Part I: systematic review and meta-analysis in diabetes management | Ann Pharmacother.; 41(10):1569-82 | Results did not separate MR from other services / interventions |
| Sketris et al. (2011) | Strategic opportunities for effective optimal prescribing and medication management | Can J Clin Pharmacol.;16(1):e103-25 | Not a systematic review |
| Bečan and Kos (2012) | Systematic review of studies evaluating benefits of clinical pharmacy and pharmaceutical care services | Farm Vestn.; 63(4):225-31. | Language |
| Maeda (2009) | Systematic review of the effects of improvement of prescription to reduce the number of medications in the elderly with polypharmacy | Yakugaku Zasshi.; 129(5):631-45 | Language |
| Castelino et al. (2009) | Targeting suboptimal prescribing in the elderly: a review of the impact of pharmacy services | Ann Pharmacother.; 43(6):1096-106 | Results did not separate MR from other services / interventions |
| Riordan et al. (2016) | The effect of pharmacist-led interventions in optimising prescribing in older adults in primary care: a systematic review | Pharmacoepidemiol Drug Saf.; 25(3): 3–680 | Not a systematic review (conference paper) |
| Riordan et al. (2016) | The effect of pharmacist-led interventions in optimising prescribing in older adults in primary care: A systematic review | SAGE Open Med.; 4: 2050312116652568 | Results did not separate MR from other services / interventions |
| Pande et al. (2013) | The effect of pharmacist-provided non-dispensing services on patient outcomes , health service utilisation and costs in low- and middle-income countries | Cochrane Database Syst Rev. 28;(2):CD010398 | MR was not evaluated |
| Colombo et al. (2017) | The effects of pharmacist interventions on adult outpatients with cancer: A systematic review | J Clin Pharm Ther.;42(4):414-24 | MR was not evaluated |
| Pen et al. (2014) | The impact of clinical pharmacy services in China on the quality use of medicines: a systematic review in context of China's current healthcare reform | Health Policy Plan.;29(7):849-72 | Results did not separate MR from other services / interventions |
| Cheema et al. (2014) | The impact of interventions by pharmacists in community pharmacies on control of hypertension: a systematic review and meta-analysis of randomized controlled trials | Br J Clin Pharmacol; 78(6):1238-47 | Results did not separate MR from other services / interventions |
| Ganguli et al. (2016) | The impact of patient support programs on adherence, clinical, humanistic, and economic patient outcomes: a targeted systematic review | Patient Prefer Adherence.;10:711-25 | MR was not evaluated |
| Meid et al. (2015) | The impact of pharmaceutical care interventions for medication underuse in older people: a systematic review and meta-analysis | Br J Clin Pharmacol.; 80(4):768-76 | Results did not separate MR from other services / interventions |
| Smith (2009) | The quality of private pharmacy services in low and middle-income countries: a systematic review | Pharm World Sci.; 31(3):351-61. | MR was not evaluated |
| Dent et al. (2007) | Tobacco interventions delivered by pharmacists: a summary and systematic review | Pharmacotherapy.; 27(7):1040-51 | MR was not evaluated |

Abbreviation: MR – Medication Review.
